# Regulation of ovarian function by growth hormone: Potential intervention of ovarian aging

**DOI:** 10.3389/fendo.2022.1072313

**Published:** 2023-01-09

**Authors:** Lei Han, Hongcheng Tian, Xiaoxiao Guo, Lei Zhang

**Affiliations:** ^1^ Department of Reproductive Medicine, Binzhou Medical University Hospital, Binzhou, Shandong, China; ^2^ Department of Reproductive Medicine, Maternal and Child Health Hospital Affiliated to Zunyi Medical University, Zunyi, Guizhou, China; ^3^ Department of Endocrinology, Binzhou Medical University Hospital, Binzhou, Shandong, China

**Keywords:** growth hormone, poor ovarian response (POR), diminished ovarian reserve (DOR), ovarian aging (OA), *in vitro* fertilization

## Abstract

Growth hormone (GH) is mainly secreted by eosinophils of anterior pituitary gland. GH plays an important role in regulating the growth and development of many tissues and cells, so it is used in the treatment of many diseases. In recent years, the regulation of GH on ovarian function has attracted much attention. GH has been applied in controlled ovarian hyperstimulation, particularly in the patients with advanced age, diminished ovarian reserve (DOR) and poor ovarian response (POR). GH can directly bind to the growth hormone receptor (GHR) on the ovary to promote the growth, maturation and ovulation of follicles, as well as to inhibit follicular atresia. GH so as to promote the occurrence of early follicles, enhance the sensitivity of follicles to gonadotropins, accelerate the maturation of oocyte nucleus, improve mitochondrial activity and the quality of oocytes through the insulin-like growth factor (IGF) system, which is an indirect regulation. The deep-seated effects of GH on human reproduction and ovarian aging need further basic research and clinical practice.

## 1 Introduction

With the development of the global economy and the transformation of women’s social roles, the childbearing age of couples around the world is increasing year by year (1, 2). The ovarian function in humans gradually declines with the increasing of age. In addition to natural aging, some patients have ovarian function decline earlier than women of the same age, and even some women’s ovaries are poor responsive to ovulation stimulants (1). How to restore the ovarian function of patients with diminished ovarian reserve (DOR), how to improve the oocyte quality of aged women, and how to enhance the ovarian response of patients with poor ovarian response (POR), have become an urgent problem in the field of assisted reproductive technology (ART).

In recent years, studies have shown that growth hormone (GH) can promote sexual maturation and gonadal cell proliferation, improve ovarian response to gonadotropins, facilitate follicular development and maturation, regulate steroid production, enhance endometrial receptivity, as well as promote embryonic quality (3). It is pointed out that GH supplementation in POR patients can significantly increase the number of oocytes retrieved, the number of embryos available for transfer and the clinical pregnancy rate (4). The combination of human menopausal gonadotrophin (HMG) and GH can significantly improve the ovarian responsiveness of patients during controlled ovarian hyperstimulation (COH), reduce the dosage of HMG, and shorten the course of treatment (5). According to the recent system review, GH reduced the dosage of gonadotropins of POR patients, and its impact on the live birth rate of this group was uncertain (3). The effect of GH on the mean number of oocytes retrieved in normal responders is uncertain, which further emphasizes the importance of GH in ovarian dysfunction.

## 2 Growth hormone and growth hormone receptor

GH is a peptide hormone mainly secreted by acidophil cells in the anterior lobe of the adenohypophysis. While reproductive cells can also secrete a small amount of GH in a continuous manner (6). GH can promote growth, regulate metabolism, and participate in the stress response of the body. The gene of human GH is located at chromosome 17 with a span of span about 66.5kbp, which can be translated into the protein containing 191 amino acids, with a relative molecular weight of 22kDa (7). There are complex mechanisms in human body to produce many forms of GH, such as polygene coding, post-translational modification, monomer polymerization, and molecular dissection. The secretion of GH is usually positively regulated by growth hormone releasing hormone (GHRH) and negatively regulated by growth hormone inhibiting hormone (GHIH) produced by the hypothalamus. GHRH is released in a pulse manner, which synchronously makes the secretion of GH in the same manner (8).

In human peripheral blood, about 45% of GH is combined with growth hormone binding protein (GHBP). GHBP can be classified into GHBP1 with high affinity and GHBP2 with low affinity (9). The former is an extracellular single chain glycoprotein generated after growth hormone receptor (GHR) hydrolysis, accounting for 85% of the total GHBP. GHBP is responsible for transporting GH to tissues, participating in the intracellular signal transduction of GH, and prolonging the half-life of GH. GH can also partly activate prolactin receptor, because GH is similar to prolactin in molecular structure (9).

GHR is a transmembrane protein in the form of heterodimers. It contains 620 amino acids, which can be expressed in reproductive system such as granulosa cells, theca cells, oocytes, cumulus cells, uterus and placenta. The human GHR gene is located at chromosome 5p12-p13.1 (10). GH has to combine with the two sub-domains of GHR on the surface of the target cell membrane respectively, and then activates intracellular downstream signal pathways through the non-receptor Try kinase pathway (11). GH may also combine with GHR, and then cut across the cytoplasm through endocytosis to enter the nucleus for direct function (10). Therefore, the quantity and function of GHR in tissues and cells affect the biological effects of GH, inducing subsequent effects of promoting synthesis and metabolism.

## 3 The direct regulation of GH on ovarian function

**Figure 1:**
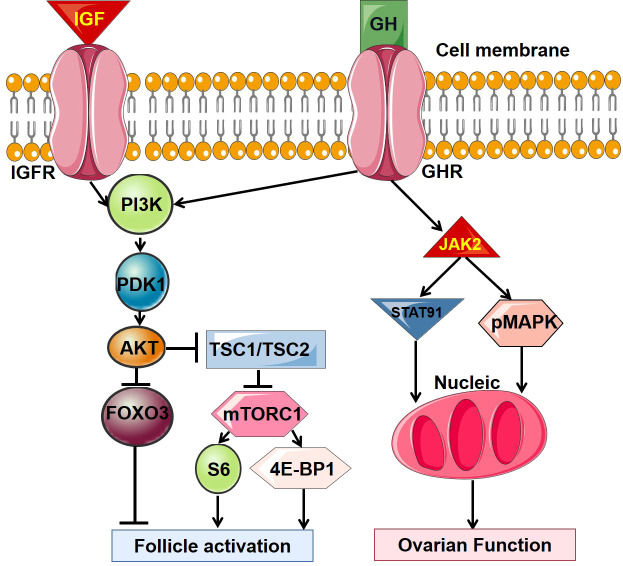
The main intracellular signal transduction pathway of GH. GH, growth hormone; GHR, growth hormone receptor; IGF, insulin-like growth factor; IGFR, insulin-like growth factor receptor.

GH can directly affect the ovarian function through GHR. It has been found that GH and GHR are expressed (protein and mRNA) in oocytes, granulosa cells and luteal cells. GHR located on the surface of oocyte membrane, cumulus cells, granulosa cells, and cumulus oocyte complexes at early follicular (12). The direct interaction between GH and GHR is mediated by cyclic adenosine monophosphate (cAMP) and cAMP dependent protein kinase A (PKA) (13). Moreover, the mitogen activated protein kinase (MAPK) and Janus Kinase (JAK) families are also involved (12). The detailed signal pathways in this regulation are summarized in **Figure 1**.

There is evidence that GH stimulates follicular growth and inhibits follicular atresia through the direct regulation. GH and GHR mRNA are detected in primordial, primary and secondary follicles of primates (14). The number of preantral follicles and antral follicles in ovaries of GHR knockout mice was significantly less than that of wild mice, suggests that GH can directly control the formation of late follicles through GHR. The addition of GH to the *in vitro* culture medium of human oocytes can significantly promote the maturation of oocytes at the germinal vesicle (GV) stage after degranulation, through accelerating meiotic progression, regulating the expression of genes related to cellular redox homeostasis, and promoting oocyte developmental potential (15). GH can also enhance the proliferation, inhibit the apoptosis and promote the expansion of cumulus cells, which secrete a variety of inhibitors to maintain meiosis of oocytes and affect oocyte nucleus maturation (16). But GH can’t completely inhibit the apoptosis of early antral follicle.

GH alone cannot promote ovulation, however, GH combined with gonadotropin can significantly improve the ovulation rate, which suggests that GH may enhance the sensitivity of gonadotropin. GH deficiency reduced the number of antral follicles and preovulatory follicles, and the number of corpus luteum (17). If GHR or GHBP gene was knocked out, the primordial and atresia follicles of mice will increase, and the primary, secondary, sinus follicles and the ovulation rate will decrease, because the formation rate of antral cavity and number of recruitable follicles decreased, and the growth and development of terminal follicles were damaged (5).

GH can also induce the production of steroid hormone directly because it can make an increase in mRNA expression of cytochrome p450 cholesterol side chain lyase, which is a rate-limiting enzyme that catalyzes the cleavage of cholesterol side chains and regulates steroid synthesis (18). If GH was injected into young experimental animals, the levels of estrogen and progesterone increased after sexual maturation (19). However, it is proved that the prolactin-like effect of GH can reduce estradiol synthesis catalyzed by follicle stimulating hormone (FSH) and increase progesterone level. This may also be due to that the regulatory effect of GH on the ovarian function depends on the stage of granulosa cells in menstrual cycle (20).

## 4 Insulin-like growth factors and indirect regulation of GH on ovarian function

GH regulates ovarian function indirectly mainly through insulin like growth factors (IGFs). IGFs are proteins with molecular weight of about 7.5 kDa, which are divided into type I and II. It is named for its high homology with insulin (21). IGFs in human peripheral circulation are mainly secreted by the liver under the induction of GH. Almost all mammalian tissues, especially ovarian granulosa and theca cells can secrete IGFs in autocrine and paracrine pathways induced by GH. IGFs are the main target of GH regulation, but also can negatively regulate GH secretion through both hypothalamus and adenohypophysis, respectively (22). IGFs act by binding to IGF receptors on the cell surface to promote the growth, development, differentiation, repairment and metabolism of cells and tissues. Insulin like growth factor binding proteins (IGFBPs) can combine with IGFs in human blood circulation and carry IGFs to specific tissues, and even regulate the interaction between IGFs and IGF receptors to affect the biological effects of IGFs (21). Interestingly, IGF receptor and IGFBP are also mainly produced by granulosa cells. IGF binds to its receptor and activates the phosphoinositol 3-kinase (PI3K)/protein kinase B (Akt) pathway, then the intracellular PIP3 pathway and MAPK pathway are activated (23). See **Figure 1** for details of signal pathways.

IGF-I and FSH in ovarian granulosa cells promote the expression of each other’s receptors, thereby mutually enhancing biological effects. After IGF-I gene of mouse was knocked out, the follicles stopped developing at the phase of preantral or early antral follicles, without ovulation (24). Therefore, IGF-I plays a starting role in the process of transforming primordial into primary follicles. In primates, IGF-II is required for the follicular FSH-dependent development. When follicles enter into the late stage of folliculogenesis, IGF bioavailability increases with the reducing of IGFBP level (21). These may allow local positive feedback loop to amplify FSH action and the expression of LH receptor, which enhances follicular responsiveness to gonadotropin and prepares for ovulation.

Exogenous GH can increase the expression level of IGF-I, IGF-I receptor and FSH receptor in granulosa cells (25). IGF-I activates JAK/STAT and ERK pathways to promote the synthesis of estradiol. On the other hand, IGF-I can also enhance the binding potential of luteinizing hormone (LH) receptor by increasing the secretion of related glycoproteins so as to increase progesterone production (5). In addition, GH/IGF-I can inhibit the activity of endogenous bone morphogenetic protein (BMP) system. The BMP system can inhibit the expression of GHR, IGF-I and IGF-I receptors in granulosa cells to weaken the biological activity of GH (26).

## 5 Regulation of GH on oocyte quality

The aneuploidy rate of oocytes is a direct manifestation of oocyte quality. Mitochondrial function determines the quality and developmental potential of oocytes. Abnormal mitochondrial function and distribution are believed to be the cause of cell aging, especially oocyte aging. GH can up-regulate and activate GHR on the surface of the membrane of human oocytes, improve mitochondrial activity (27). Mitochondria are essential elements for energy supply in the process of follicular development, oocyte spindle formation, chromosome separation, meiosis, fertilization, etc. Thus, mitochondrial function directly affects the generation of oocyte aneuploidy during meiosis and interferes with oocyte quality (28). Previous studies found that the number of functional mitochondria and the content of mitochondrial DNA (mtDNA) were significantly reduced in oocytes of patients with POI, DOR, POR and advanced age, accompanied by down-regulation of GHR expression and failure of fertilization (29). After GH treatment, the number of GHR and mitochondria in oocytes of these patients are up-regulated significantly. The outcome indicators of these patients when receiving assisted reproductive technology also improved accordingly. Besides, the content of cumulus granulosa cell mtDNA may also be related to oocyte quality and embryo implantation potential (20). GH can improve the number of retrieved oocytes, embryo implantation rate, clinical pregnancy rate, live birth rate and mtDNA content in cumulus granulosa cells of patients with normal ovarian reserve but low rate of high-quality embryos (29).

The reactive oxygen species (ROS) produced by oocytes under oxidative stress can lead to oxidative damage to cells, especially to nucleus and DNA (30). DNA damage and nuclear genome mutation are both important mechanisms leading to oocyte aging. Aging will also aggravate ROS production and mtDNA damage. Animal studies showed that GH could significantly reduce the expression of γ-H2AX and apoptosis of aging oocytes to improve the ovarian reserve and oocyte quality (27). Compared with the control group, POR patients who received GH treatment had significantly lower oxidative stress level in follicular fluid and granulosa cells, and significantly increased the number of high-quality embryos and clinical pregnancy rate. It was speculated that reducing oxidative stress might be one of the mechanisms of GH improving oocyte quality (20).

## 6 Dosage of GH and ovarian response

Clinical study found that only increasing the dosage of gonadotropin in the COH process of patients with POR and DOR could not promote ovarian response. Some of the patients may be due to the lack of FSHR or functional mitochondria in granulosa cells (20). IGF-I is known to increase the expression of FSHR mRNA in granulosa cells to enhance the stimulating effects of FSH, which indicates that GH may promote ovarian response mainly through indirect regulation (31). There are few reports on the correlation between GHR and the dosage of gonadotropin, and the role of GH needs to be further studied.

Adding a certain amount of GH during COH process of patients with POR can improve ovarian response, increase the number of follicles, and reduce the amount of gonadotropin (32). The GH level in follicular fluid is usually proportional to ovarian response, but it is inversely proportional to gonadotropin dosage (24). However, the survival rate of follicles cultured in medium with extremely high concentration (100mg/ml) of GH is significantly lower than the lower groups. Thus the development of follicles requires an appropriate amount of GH, and too high or too low concentration of GH is not conducive to the growth of follicles (16).

So far, in most of the RCT studies about the relationship between GH supplementation and the improvement of IVF assisted pregnancy outcome in infertile women, the starting time of drug use is mostly within the ovulation induction cycle or one week ahead, and a few studies are 2 to 6 weeks before ovulation induction (33). The GH dose of most studies is low, such as 2-7.5IU, and only a few studies use moderate and high dose of 12-24IU. Existing meta-analyses all believe that the use of GH in the ovulation induction cycle can increase the number of oocytes retrieved, the number of oocytes in MII phase, the clinical pregnancy rate and the live birth rate of POR, POI or elderly patients, or improve several of these outcome indicators. The recommended dose of GH is low to medium dose, 4IU-12IU per day (34, 35).

The medium dose of GH pretreatment to elderly experimental animals for 8 weeks in advance can improve oocyte quality by improving its mitochondrial membrane potential, distribution, and increase the number of retrieved oocytes and MII egg rate in COH. The starting time of GH usage in most RCT studies is the 21st day of the previous menstrual cycle or the first day of the COH cycle, and the stop time the HCG-Day (33, 34). Theoretically speaking, GH should be used 3 months before the human COH cycle, so that GH can regulate the follicular development process from preantral to mature stage, which may improve the quality of oocytes completely. Of course, the use of GH should also be considered comprehensively according to the patient’s acceptability, economic status and other factors.

Some studies have shown that although GH can reduce the dosage of Gn, shorten the duration of COH, increase the number of oocytes and transferable embryo, it does not significantly increase the clinical pregnancy and live birth rate (2, 33). Therefore, the popularization value of GH in assisted reproductive therapy needs more careful analysis. In the future, higher quality, larger population, multi-center clinical research and more in-depth *in vivo* and *in vitro* research are needed to explore and verify the role of GH in the COH cycle.

## 7 Safety of GH treatment

Previous studies on the adverse reactions after long-term GH treatment mainly focused on mortality, new diabetes, and the occurrence of malignant tumors and thyroid diseases (36). In the past, GH was believed to have potential carcinogenicity because of the animal experiments that GH gene transfected endometrial cancer cells progressed more rapidly in nude mice than those without GH transfected. Many studies have proved that long-term GH treatment does not increase the risk of malignant tumors. On the contrary, GH has a positive effect on cardiovascular disease, stroke and fracture (37). But the analysis showed that GH pretreatment in IVF treatment did not increase the incidence of adverse events. GH can lead to significant metabolic changes by increasing cholesterol and interfering with renin angiotensin (38). Although there is no definite evidence about carcinogenesis of GH in therapeutic dose, the safety of long-term drug use in patients with malignant tumor, aggravating metabolic disorder, and other unforeseen adverse events should be continuously monitored (39, 40). It has not been found that the application of GH in IVF treatment will cause adverse effects on hysteromyoma, endometriosis, adenomyosis, adverse pregnancy outcomes, etc., but long-term follow-up and observation are still needed.

## 8 Summary

The basic researches on GH regulating ovarian function have laid a full theoretical foundation for its clinical application. GH plays an important role in follicular growth and development, follicular maturation, ovulation, oocyte quality and steroid hormone synthesis. The use of GH in IVF treatment can improve the outcome of assisted reproductive therapy in patients with POR, DOR and advanced age. Although there are still some disputes about increasing the clinical pregnancy rate and live birth rate. The previous clinical studies reached few precise conclusions on GH, so further research is needed to solve such problems about suitable population, starting time, dosage and course of treatment. In consideration of potential risks, patients receiving treatment with assisted reproductive technology should conduct individualized comprehensive evaluation before using GH, and then carefully formulate treatment plans for patients.

## Author contributions

LH and LZ determine the topic and write the manuscript, HT and XG retrieve literatures and analyze data. All authors contributed to the article and approved the submitted version.
